# The impact of badminton participation on attention control in college students: the mediating role of flow experience

**DOI:** 10.3389/fpsyg.2025.1738888

**Published:** 2026-01-08

**Authors:** Yawei Ren, Weisong Chen, Bo Peng, Lishan Yu, Mingmin Kong

**Affiliations:** 1 School of Sports Training, Chengdu Sport University, Chengdu, Chengdu, Sichuan, China; 2 Physical Education, Sichuan Normal University, Chengdu, Sichuan, China

**Keywords:** attentional control, badminton participation, college students, flow experience, mental concentration, perceived control, perceived enjoyment

## Abstract

**Purpose:**

This study examined whether badminton participation predicts attentional control in college students and whether three dimensions of flow experience—perceived control, perceived enjoyment, and mental concentration—mediate this association.

**Methods:**

A total of 762 college students who regularly engaged in badminton completed standardized questionnaires assessing badminton participation, flow experience, and attentional control. Structural equation modeling with a bias-corrected bootstrap procedure (5,000 resamples) was used to test the hypothesized direct and indirect pathways.

**Results:**

Badminton participation was positively associated with attentional control (*β* = 0.39, *p* < 0.001). Flow experience partially mediated this relationship. The indirect effects through perceived control (*β* = 0.12, *p* = 0.004), perceived enjoyment (*β* = 0.08, *p* = 0.012), and mental concentration (*β* = 0.10, *p* = 0.006) were significant, accounting for 17.39, 11.59, and 14.49% of the total effect, respectively.

**Conclusion:**

Badminton participation appears to improve attentional control both directly and by enhancing flow experience. These findings clarify the psychological mechanisms linking sport participation and attentional regulation and provide empirical support for incorporating structured badminton activities into collegiate physical education and cognitive training programs aimed at strengthening attentional stability.

## 1 Introduction

With the rapid pace of modern society and the increasing pressure of daily life, individuals face unprecedented challenges in managing and focusing their attention. Especially in learning, work, and daily life, many people frequently experience difficulty concentrating or excessive distraction, leading to inefficiency and increased psychological stress. Attention control refers to an individual’s ability to effectively manage and adjust their attention resources in complex environments to achieve goal-directed behavior (Eysenck et al., 2007). Efficient attention control is crucial for academic performance, work efficiency, and psychological well-being (Cardoso-Leite et al., 2021; Hudson et al., 2024).

Badminton, as a high-intensity sport, combines competitiveness, reaction speed, and physical coordination. It requires athletes to make rapid reactions and precise decisions within a short period while maintaining high levels of attention. This not only enhances physical fitness but has also been proven to improve attention control and cognitive functions during physical activity (Oishi and Yamasaki, 2024; Cabello-Manrique et al., 2022). Additionally, hitting the shuttlecock in different directions, at varying heights and speeds, can increase the excitability and sensitivity of the nervous system, enhancing the number of muscle fibers involved in the sport. These characteristics have been shown to improve the executive functions and physical capabilities of long-term badminton players (Oh et al., 2015). For instance, compared to tennis and table tennis, badminton’s shuttlecock speed variation is faster and more unpredictable, which places higher demands on athletes’ reaction speed and attention control (Nikolakakis and Telopoulos, 2023; Kaplan et al., 2019).

However, while research has indicated that physical activity positively impacts cognitive functions (Zhang et al., 2023; Garrett et al., 2024; Liu et al., 2025) and that badminton’s cognitive load characteristics help enhance attention control (van de Water et al., 2017), existing literature mainly focuses on the direct relationship between exercise intensity and attention (Sanchis-Navarro et al., 2024; Pindus et al., 2023), with limited exploration of the psychological mechanisms involved in the exercise process. In particular, the role of flow experience as a mediator between exercise participation and cognitive enhancement has not been fully addressed. Flow experience, as a deep psychological state, refers to a condition where an individual becomes fully immersed in an activity, exhibiting high concentration and automatic responses (Csikszentmihalyi, 1990). Existing research has shown that flow experience can enhance an individual’s focus and emotional stability, thereby improving cognitive functions (Tavares and Freire, 2016). Furthermore, flow experience has been shown to be closely related to sports performance and cognitive function (Eschmann et al., 2022; Harris et al., 2021), but most studies focus on aerobic exercise or single sports, lacking a systematic analysis of flow experience in high-intensity, fast-paced sports like badminton.

Based on this, this study aims to explore the relationship between badminton participation and attention control, and further analyze the mediating role of flow experience in this relationship. Specifically, this study will focus on the different dimensions of badminton participation (such as reaction speed, attention demands, decision-making time limits, etc.) and their impact on attention control. Additionally, the study will examine the mediating role of flow experience between exercise participation and attention control. By employing surveys and structural equation modeling, the study aims to address the following core questions: (1) Is badminton participation related to improvements in attention control? (2) Does flow experience mediate the relationship between badminton participation and attention control? The results of this study will not only help deepen the understanding of the relationship between exercise participation and cognitive enhancement, but also provide new theoretical support for exercise interventions and cognitive training.

## 2 Literature review and hypotheses

### 2.1 The relationship between badminton participation and cognitive function

Badminton is a sport that requires high levels of concentration, rapid reactions, and complex motor coordination. With the development of sports science, more and more research has focused on the relationship between exercise and cognitive function, particularly its impact on attention control (Liu et al., 2025; Blomstrand et al., 2023). Regular physical exercise has been shown to have a significant positive impact on cognitive function, especially on attention control (He et al., 2025; Lin et al., 2025). Research indicates that badminton, as a high-intensity sport, can significantly improve cognitive processing abilities, especially in terms of reaction time, concentration, and cognitive flexibility (Wang et al., 2023).

What makes badminton unique is that it not only requires excellent physical fitness and reaction skills, but also demands continuous precise judgments and rapid reactions, while maintaining high levels of attention in complex situations (Xie and Yu, 2014). The rapid changes in the court, variation in shuttlecock speed, and the need to quickly judge an opponent’s intentions during badminton matches promote greater attention focus and psychological regulation during the game (Pan et al., 2016). Compared to other high-intensity sports such as table tennis or tennis, badminton presents more significant challenges in terms of reaction time, decision complexity, and the integration of physical and reaction demands (Nikolakakis and Telopoulos, 2023; Phomsoupha and Laffaye, 2015). However, existing literature rarely explores how badminton improves attention control through psychological mechanisms, particularly the mediating role of flow experience.

### 2.2 The psychological mechanism and influencing factors of attention control

Attention control refers to an individual’s ability to effectively allocate and adjust attention resources when faced with multiple tasks or distractions (Eysenck et al., 2007). According to executive function theory, attention control is one of the core components of executive function, directly influencing an individual’s decision-making, learning, and task performance in complex situations (Miyake et al., 2000). Several studies have shown that exercise, particularly high-intensity competitive sports, can significantly increase brain blood flow and promote the secretion of neurotrophic factors, thereby enhancing neural connections and improving cognitive functions, especially attention and working memory (Liu et al., 2023; Nilsson et al., 2020).

Badminton, as a high-intensity reactive sport, requires athletes to make quick decisions and judgments in complex situations, which aligns with attention resource allocation theory (Hübner and Bennemann, 1973). This theory suggests that individuals have limited attention resources, and how these resources are efficiently distributed among multiple tasks directly affects task completion efficiency (MacKay-Brandt, 2011; Lee et al., 2025). The rapid changes and decision-making demands in badminton can enhance individuals’ focus and resource management abilities, thereby improving their attention control (Takahashi and Grove, 2019; Wang et al., 2023).

### 2.3 The theory and research progress of flow experience

Flow experience, proposed by Csikszentmihalyi, refers to a psychological state in which an individual becomes fully immersed in an activity due to a high match between the challenges of the activity and the individual’s skills, resulting in a pleasurable, highly focused state (Tavares and Freire, 2016). In a flow state, individuals exhibit high concentration on the activity, losing awareness of time and external factors, and experiencing a sense of fulfillment and self-realization (Isham and Jackson, 2022). Self-determination theory emphasizes that individuals are more likely to enter a flow state under intrinsic motivation, particularly in situations where challenges match abilities (Ryan and Deci, 2000). Flow experience not only contributes to improved sports performance but also positively affects cognitive functions and psychological states. Research shows that flow experience can enhance focus, emotional regulation, and executive function (Golub et al., 2016; Ross and MacIntyre, 2020; Xie, 2022). In the context of sports, flow states can increase an individual’s focus on the activity and enhance their participation, thereby improving sports performance (Jackson and Marsh, 1996; Swann et al., 2012). For sports like badminton, which require rapid reactions and high attention, flow experience is particularly important. Flow allows individuals to focus more on the sport, reduce external distractions, and improve attention control and performance.

### 2.4 The role of flow experience in sports participation and cognitive enhancement

The role of flow experience in sports has gradually gained attention from researchers, especially in high-intensity sports. Flow states help athletes concentrate their attention and make quick responses, thus enhancing performance (Swann et al., 2017; Schuler and Brunner, 2009). In reactive sports like badminton, athletes often enter a flow state due to the high level of challenge and skill matching, which improves their attention control and executive function (Stavrou et al., 2007; Harris et al., 2017).

Research indicates that flow experience can enhance sports performance and cognitive function, especially when athletes are in a highly focused state, enabling them to more efficiently manage cognitive resources, concentrate attention on important information, and avoid distractions (Hübner and Bennemann, 1973). Therefore, flow experience may play a mediating role between badminton participation and attention control.

Based on the above literature review, the objectives and hypotheses of this study are as follows.

**Figure 1:**
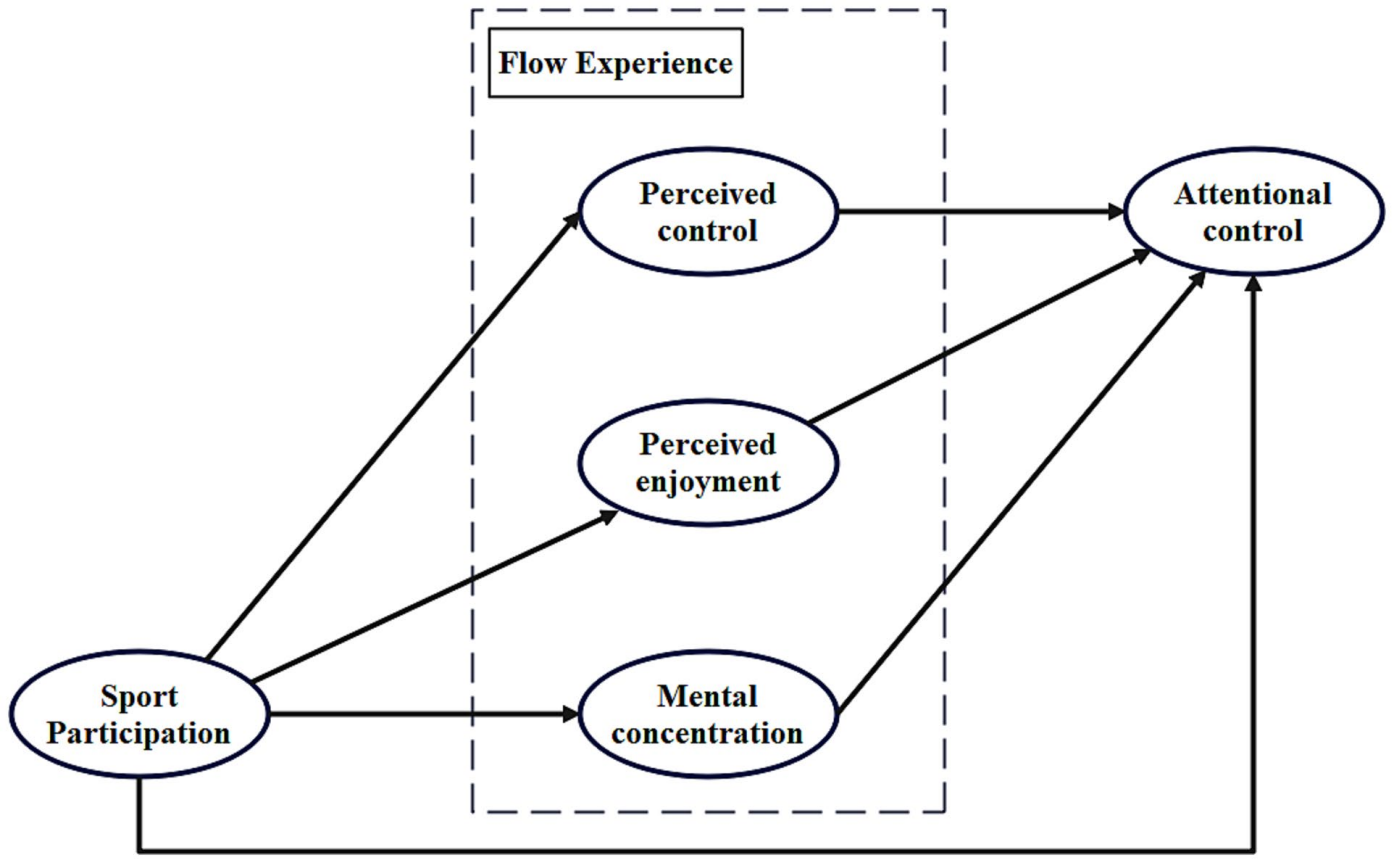
Conceptual model of the impact of badminton participation on attentional control among college students based on flow experience.

This study primarily uses a cross-sectional survey to explore whether flow experience mediates the effect of badminton participation on college students’ attention control. As a high-intensity sport, badminton requires athletes to have quick reactions, precise decision-making, and a high level of concentration, which makes it particularly suitable for studying attention control. The main research question is: How does college students’ participation in badminton influence their attention control through flow experience? As shown in Figure 1, the main hypotheses of the study are:

*H1*: Participation in badminton has a positive effect on college students’ attention control.

*H2*: Perceived control mediates the relationship between participation in badminton and college students’ attention control.

*H3*: Perceived enjoyment mediates the relationship between participation in badminton and college students’ attention control.

*H4*: Mental concentration mediates the relationship between participation in badminton and college students’ attention control.

## 3 Materials and methods

### 3.1 Participants and data collection

This study adopted a stratified random sampling method. Undergraduate students from five provinces and municipalities in China—Sichuan, Shanghai, Guangdong, Chongqing, and Yunnan—were recruited, covering first- to fourth-year students aged between 18 and 24 years.

According to general empirical guidelines in social science research, the recommended sample size is typically 10 to 15 times the total number of questionnaire items (Jeffrey and Hoogland, 1998). The measurement tools used in this study included the Sport Participation Scale (20 items), Flow Experience Scale (10 items), and Attentional Control Scale (20 items), with a total of 50 items. Based on this criterion, the required sample size was estimated to range from 500 to 750 participants.

Data were collected from March 3 to August 31, 2025. All participants completed the same Chinese versions of the questionnaires under standardized administration procedures across the five regions to ensure comparability. A total of 900 questionnaires were distributed, and 762 valid responses were obtained, yielding a valid response rate of 84.67%. During data screening, questionnaires with missing data, inconsistent answers, or fixed-pattern responses were excluded to ensure the quality of the dataset.

### 3.2 Measurement instruments

#### 3.2.1 Sport participation

This study uses the Exercise Participation Scale developed by Zheng et al. (2023) to assess exercise participation. The scale includes seven dimensions: functional evaluation, exercise attention focus, exercise ability, learning behavior, exercise behavior, exercise-related emotions, and leisure-related emotions, with a total of 20 items. The scale aims to assess college students’ frequency of participation, duration, and their subjective perception of physical and mental health during exercise. The scale uses a 5-point Likert scale (1 = “Strongly Disagree,” 5 = “Strongly Agree”), with higher scores indicating higher levels of exercise participation. Previous studies have shown that the scale has good reliability and validity (Cronbach’s *α* > 0.80). In this study, the Cronbach’s *α* coefficient of the Exercise Participation Scale is 0.92, indicating high internal consistency and good measurement reliability.

#### 3.2.2 Flow experience

This study employed the Flow Experience Scale developed by Zhang (2020) to measure college students’ flow states during badminton participation. The scale consists of three dimensions—perceived control, perceived enjoyment, and mental concentration—with a total of 10 items. These dimensions, respectively, capture individuals’ cognitive sense of mastery and control, affective pleasure and intrinsic satisfaction, and the intensity and continuity of attentional focus experienced during exercise. Responses were rated on a 5-point Likert scale (1 = “Strongly Disagree,” 5 = “Strongly Agree”), with higher scores indicating higher levels of flow experience. Previous research has demonstrated that the scale possesses satisfactory psychometric properties across diverse cultural and sport contexts (Cronbach’s *α* > 0.80). In the present study, the internal consistency coefficients for each subscale were 0.91 for perceived control, 0.81 for perceived enjoyment, and 0.79 for mental concentration, all exceeding the recommended threshold of 0.70. These results indicate high internal consistency and reliability, confirming that the scale effectively captured the multidimensional structure of flow experience among college students during badminton participation.

#### 3.2.3 Attentional control

This study uses the Attention Control Scale developed by Derryberry and Reed (2002) to assess college students’ attention control levels. The scale includes two dimensions: attention focusing and attention shifting, with a total of 20 items. The scale aims to comprehensively assess an individual’s ability to maintain attention focus and flexibly shift attention during task performance. The scale uses a 5-point Likert scale (1 = “Strongly Disagree,” 5 = “Strongly Agree”), with higher scores indicating stronger attention control abilities. Cross-cultural studies have shown that the scale has good reliability and validity (Cronbach’s *α* > 0.80). In this study, the Cronbach’s *α* coefficient of the Attention Control Scale is 0.86, indicating high internal consistency, stable and reliable measurement results, and the ability to effectively reflect college students’ attention control levels influenced by badminton participation and flow experience.

### 3.3 Data analysis

All statistical analyses were conducted using IBM SPSS Statistics 26.0 and AMOS 26.0. Prior to analysis, the dataset was screened for missing values, outliers, and approximate normality to ensure data quality. Given the large sample size (*N* = 762) and prior evidence that parametric procedures such as regression and SEM are generally robust to moderate departures from normality in large samples, the data were deemed suitable for normal-theory analyses.

Descriptive statistics, including means, standard deviations, and frequency distributions, were computed to summarize the demographic characteristics and key study variables. The internal consistency of each scale was examined using Cronbach’s *α*, with values above 0.70 considered acceptable (Ahmad et al., 2024). To further assess measurement reliability and convergent validity, composite reliability (CR) and average variance extracted (AVE) were calculated. The minimum acceptable thresholds were set at CR > 0.70 and AVE > 0.50, while standardized factor loadings greater than 0.60 were regarded as adequate (Cheung et al., 2024).

The measurement validity of the latent constructs—sport participation, perceived control, perceived enjoyment, mental concentration, and attentional control—was examined through confirmatory factor analysis (CFA). Model fit was evaluated using multiple indices, including the ratio of chi-square to degrees of freedom (*χ*^2^/df < 5), comparative fit index (CFI > 0.90), Tucker–Lewis index (TLI > 0.90), standardized root mean square residual (SRMR < 0.08), and root mean square error of approximation (RMSEA < 0.08) with its 90% confidence interval (Hair, 2011). To clarify measurement validity across regions, a series of multi-group CFAs were conducted to test configural, metric, and scalar invariance of the measurement model across the different provincial samples.

To assess potential common method bias, both Harman’s single-factor test and a series of alternative CFA models were performed. The absence of serious bias was confirmed if (a) the first unrotated factor explained less than 40% of the total variance and (b) the hypothesized multi-factor model demonstrated substantially better fit than the single-factor model (Kock et al., 2021).

After establishing reliability and validity, Pearson correlation analysis was conducted to examine the bivariate relationships among the key variables (Tang and Wen, 2020). Before running the regression models, multicollinearity diagnostics were computed for all predictors using variance inflation factors (VIF). All VIF values were below 5, indicating no problematic multicollinearity among the predictors. Hierarchical multiple regression analyses were then performed to test the direct and mediating effects of sport participation and flow experience on attentional control. Statistical significance was evaluated at *p* < 0.05, *p* < 0.01, and *p* < 0.001.

To verify the structural relationships among the constructs, structural equation modeling (SEM) was conducted in AMOS 26.0. The hypothesized model tested both direct and indirect paths from badminton participation to attentional control via flow experience. Model adequacy was assessed using the same fit indices as described above.

Finally, the mediating effects of perceived control, perceived enjoyment, and mental concentration were tested using a bias-corrected percentile bootstrap method with 5,000 resamples. Mediation was considered significant when the 95% confidence interval (CI) for the indirect effect did not include zero (Preacher and Hayes, 2008).

## 4 Result

### 4.1 Sample characteristics

A total of 762 valid responses were obtained. Table 1 displays the demographic characteristics of the participants. Among the respondents, 400 (52.49%) were male and 362 (47.51%) were female, showing a relatively balanced gender composition. Regarding academic year, sophomores constituted the largest group (38.85%), followed by freshmen (22.05%), juniors (19.55%), and seniors (19.55%). In terms of major type, 370 participants (48.56%) were from sports-related majors, while 392 (51.44%) were from non-sports majors. Overall, the sample demonstrated a well-distributed representation across demographic categories.

**Table 1:** Demographic characteristics of the sample (*N* = 762).

Variable	Category	Frequency	Percentage (%)
Gender	Male	400	52.49%
	Female	362	47.51%
Academic year	Freshman	168	22.05%
	Sophomore	296	38.85%
	Junior	149	19.55%
	Senior	149	19.55%
Major	Sports-related	370	48.56%
	Non-sports-related	392	51.44%

### 4.2 Descriptive statistics and measurement reliability

Table 2 presents the descriptive statistics, internal consistency reliability, and confirmatory factor analysis (CFA) results for the main variables. The internal consistency reliability of all scales was satisfactory, with Cronbach’s alpha (*α*) values ranging from 0.79 to 0.92, exceeding the recommended threshold of 0.70, thus demonstrating good internal reliability. The factor loadings for all measurement items ranged between 0.65 and 0.86, meeting the acceptable standard of 0.60 or higher, which indicates adequate convergent validity. The composite reliability (CR) values for the constructs ranged from 0.79 to 0.91, surpassing the minimum criterion of 0.70, further confirming the reliability of the measurement model. The average variance extracted (AVE) values ranged from 0.53 to 0.66, all exceeding the 0.50 benchmark, indicating that the latent variables explained more than half of the variance of their observed indicators. Collectively, these results support the reliability and convergent validity of the measurement instruments employed in this study.

**Table 2:** Descriptive statistics, internal consistency reliability, and fit indices for confirmatory factor analysis (CFA) of key variables.

Variable	Mean	SD	*α*	Factor loading	CR	AVE
Sport participation	3.07	0.53	0.92	0.68–0.83	0.79	0.56
Perceived control	3.68	0.90	0.91	0.73–0.78	0.91	0.56
Perceived enjoyment	3.65	0.85	0.81	0.73–0.84	0.82	0.61
Mental concentration	3.84	1.02	0.79	0.78–0.84	0.79	0.66
Attentional control	3.55	0.62	0.86	0.65–0.86	0.91	0.53

SD, standard deviation; *α*, Cronbach’s alpha; CR, composite reliability; AVE, average variance extracted.

### 4.3 Common method bias test

To evaluate the potential influence of common method bias, both Harman’s single-factor test and a series of confirmatory factor analyses (CFAs) were performed. The unrotated exploratory factor analysis yielded seven factors with eigenvalues greater than 1, explaining a total of 62.53% of the variance. The first factor accounted for 29.35% of the total variance, which is well below the critical threshold of 40%. This result indicates that no single factor dominated the variance, suggesting that common method bias was unlikely to pose a substantial threat to the validity of the findings.

To further confirm this result, alternative measurement models were tested through CFAs. As shown in Table 3, the one-factor model demonstrated poor fit to the data (*χ*^2^/df = 10.62, CFI = 0.787, TLI = 0.759, RMSEA = 0.112). Model fit improved progressively as the number of latent factors increased. The five-factor model, corresponding to the hypothesized structure comprising sport participation, perceived control, perceived enjoyment, mental concentration, and attentional control, exhibited an excellent fit to the data [*χ*^2^/df = 1.34, CFI = 0.993, TLI = 0.992, SRMR = 0.024, RMSEA = 0.021 (90% CI: 0.011–0.029)]. These results provide strong evidence that common method variance was not a serious concern in this study and that the constructs were empirically distinct.

**Table 3:** Fit indices for alternative measurement models.

Model	*χ*^2^/df	CFI	TLI	SRMR	RMSEA (90% CI)
One-factor	10.62	0.787	0.759	0.083	0.112 (0.107, 0.118)
Two-factor	10.07	0.801	0.773	0.081	0.109 (0.104, 0.115)
Three-factor	8.21	0.844	0.819	0.076	0.097 (0.092, 0.103)
Four-factor	4.40	0.928	0.915	0.058	0.067 (0.061, 0.073)
Five-factor	1.34	0.993	0.992	0.024	0.021 (0.011, 0.029)

### 4.4 Correlation analysis and discriminant validity

Table 4 presents the correlations among the key study variables as well as the results of the discriminant validity test. The correlations between variables were all positive and statistically significant (*p* < 0.001), with coefficients ranging from 0.29 to 0.52. Sport participation showed moderate correlations with the three dimensions of flow experience—perceived control (*r* = 0.40), perceived enjoyment (*r* = 0.29), and mental concentration (*r* = 0.33)—and with attentional control (*r* = 0.39). Similarly, the flow dimensions were moderately correlated with each other (*r* = 0.48–0.52) and with attentional control (*r* = 0.40–0.45), indicating that while the constructs are related, they remain conceptually distinct.

**Table 4:** Correlations and discriminant validity among key variables.

Variable	Sport participation	Perceived control	Perceived enjoyment	Mental concentration	Attentional control
Sport participation	0.75				
Perceived control	0.40^***^	0.75			
Perceived enjoyment	0.29^***^	0.52^***^	0.78		
Mental concentration	0.33^***^	0.49^***^	0.48^***^	0.81	
Attentional control	0.39^***^	0.45^***^	0.40^***^	0.41^***^	0.73

^***^p < 0.001. Values on the diagonal represent the square roots of the average variance extracted (AVE).

The square roots of the AVE values, ranging from 0.73 to 0.81, all exceeded the corresponding inter-construct correlations. This finding indicates satisfactory discriminant validity, confirming that each construct was empirically distinct.

### 4.5 Multiple regression analysis

Table 5 reports the results of hierarchical multiple regression analyses conducted to examine the predictors of attentional control and to provide preliminary evidence for subsequent mediation testing. Prior to estimating the models, multicollinearity diagnostics indicated no serious concerns, with all variance inflation factor (VIF) values below 5.

**Table 5:** Multiple regression analysis of predictors of attentional control.

Variables	Model 1 (controls only)		Model 2 (+sport participation)		Model 3 (+flow experience)	
	*β*	SE	*β*	SE	*β*	SE
Control variables						
Gender	−0.06	0.05	−0.12^***^	0.04	−0.13^***^	0.04
Academic year	−0.11^**^	0.02	−0.05	0.02	0.00	0.02
Major	0.03	0.04	0.00	0.04	0.04	0.04
Independent variable						
Sport participation			0.40^***^	0.04	0.23^***^	0.04
Mediator						
Perceived control					0.22^***^	0.03
Perceived enjoyment					0.16^***^	0.03
Mental concentration					0.16^***^	0.02
Model summary						
*R* ^2^	0.02		0.17		0.32	
Δ*R*^2^	—		0.15^***^		0.15^***^	
*F*	4.57^**^		39.19^***^		51.82^***^	

Standardized coefficients (*β*) are reported. ^*^p < 0.05, ^**^p < 0.01, and ^***^p < 0.001.

In Model 1, demographic variables were included as controls. Gender (*β* = −0.06, *p* > 0.05) and major (*β* = 0.03, *p* > 0.05) were not significant predictors, whereas academic year exerted a small but significant negative effect (*β* = −0.11, *p* < 0.01). This model accounted for 2% of the variance in attentional control (*R*^2^ = 0.02, *F* = 4.57, *p* < 0.01).

In Model 2, sport participation was added as an independent variable. The result indicated a significant positive association with attentional control (*β* = 0.40, *p* < 0.001). The explained variance increased from 2 to 17% (Δ*R*^2^ = 0.15, *p* < 0.001), suggesting that sport participation contributed meaningfully to attentional control beyond demographic factors.

In Model 3, the three dimensions of flow experience—perceived control, perceived enjoyment, and mental concentration—were simultaneously entered. All three dimensions exhibited significant positive effects: perceived control (*β* = 0.22, *p* < 0.001), perceived enjoyment (*β* = 0.16, *p* < 0.001), and mental concentration (*β* = 0.16, *p* < 0.001). After inclusion of these variables, the coefficient of sport participation decreased from *β* = 0.40 (*p* < 0.001) to *β* = 0.23 (*p* < 0.001), while the model’s explanatory power increased to *R*^2^ = 0.32 (Δ*R*^2^ = 0.15, *p* < 0.001).

These results provide preliminary empirical support for the hypothesized mediating role of flow experience in the relationship between sport participation and attentional control.

### 4.6 Structural equation modeling analysis

The hypothesized structural model was examined using structural equation modeling (SEM). The model demonstrated a satisfactory overall fit to the data: *χ*^2^/df = 3.16, CFI = 0.955, TLI = 0.946, SRMR = 0.067, and RMSEA = 0.053 (90% CI (0.048, 0.059)]. All fit indices met the recommended thresholds [*χ*^2^/df < 5, CFI and TLI > 0.90, SRMR < 0.08, RMSEA < 0.08), indicating that the proposed model provided an adequate representation of the observed data.

**Figure 2:**
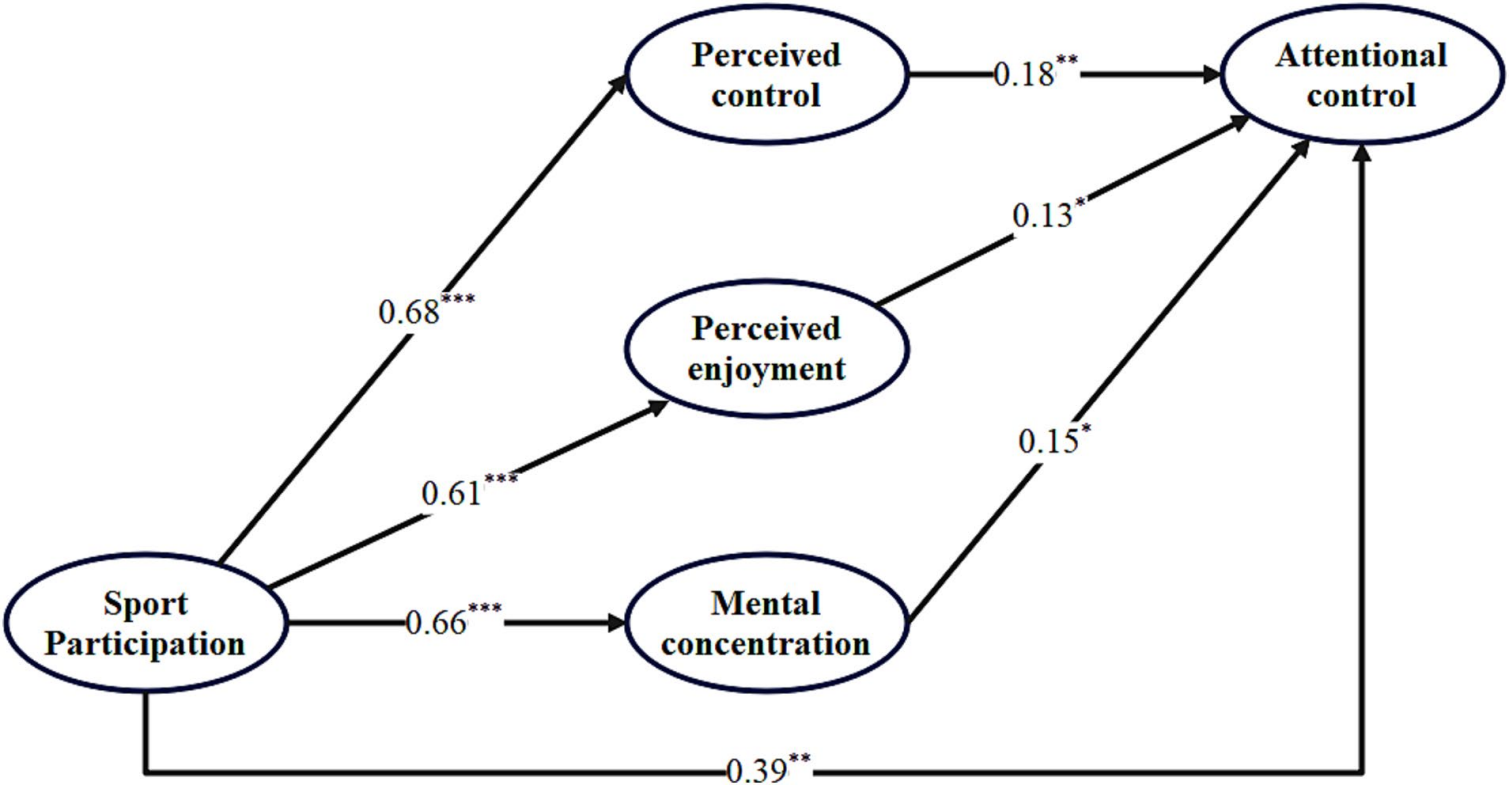
Standardized path coefficients of the structural model. ^*^p < 0.05, ^**^p < 0.01, and ^***^p < 0.001.

As shown in Figure 2, sport participation exerted significant positive effects on perceived control (*β* = 0.68, *p* < 0.001), perceived enjoyment (*β* = 0.61, *p* < 0.001), and mental concentration (*β* = 0.66, *p* < 0.001). In turn, perceived control (*β* = 0.18, *p* < 0.01), perceived enjoyment (*β* = 0.13, *p* < 0.05), and mental concentration (*β* = 0.15, *p* < 0.05) each had significant positive effects on attentional control. Sport participation also retained a significant positive direct effect on attentional control (*β* = 0.39, *p* < 0.01), suggesting a pattern of partial mediation.

### 4.7 Bootstrapping mediation test

To further examine the mediating effects of the three flow experience dimensions, a bootstrapping procedure with 5,000 resamples and bias-corrected 95% confidence intervals (CIs) was conducted. The total, direct, and indirect effects are summarized in Table 6.

**Table 6:** Total, direct, and indirect effects in the multiple mediator model.

Path	*β*	Boot SE	*p*	Boot LLCI	Boot ULCI	Ratio
Direct effect						
Sport participation → Attentional control	0.39	0.13	<0.001	0.19	0.71	56.52%
Indirect effects						
Sport participation → Perceived control → Attentional control	0.12	0.05	0.004	0.10	0.22	17.39%
Sport participation → Perceived enjoyment → Attentional control	0.08	0.04	0.012	0.05	0.16	11.59%
Sport participation → Mental concentration → Attentional control	0.10	0.05	0.006	0.07	0.19	14.49%
Total effect	0.69	0.05	<0.001	0.60	0.79	100%

Boot LLCI, the lower bound of the 95% confidence interval. Boot ULCI, the upper limit of the 95% confidence interval (percentile bootstrap method with bias correction). The bootstrap sample size is set at 5,000.

The total effect of sport participation on attentional control was significant [*β* = 0.69, *p* < 0.001, 95% CI (0.60, 0.79)], supporting the overall predictive relationship between the two variables. Consistent with *H1*, the direct effect of sport participation on attentional control remained significant [*β* = 0.39, *p* < 0.001, 95% CI (0.19, 0.71)], accounting for 56.52% of the total effect.

Regarding the mediating effects, all three dimensions of flow experience showed significant indirect effects. Specifically, the indirect effect through perceived control [*β* = 0.12, *p* = 0.004, 95% CI (0.10, 0.22)] supported *H2*, the indirect effect through perceived enjoyment [*β* = 0.08, *p* = 0.012, 95% CI (0.05, 0.16)] supported *H3*, and the indirect effect through mental concentration [*β* = 0.10, *p* = 0.006, 95% CI (0.07, 0.19)] supported *H4*. The respective proportions of the total effect explained by these mediating paths were 17.39, 11.59, and 14.49%.

The bias-corrected bootstrap confidence intervals for all indirect effects did not include zero, indicating that the mediating effects of perceived control, perceived enjoyment, and mental concentration were statistically significant. Collectively, these results confirmed partial mediation, demonstrating that the influence of sport participation on attentional control operated both directly and indirectly through the multidimensional structure of flow experience.

## 5 Discussion

### 5.1 Overview of the findings

This study investigated the relationship between badminton participation and attentional control among college students, with a particular focus on the mediating role of flow experience and its three dimensions: perceived control, perceived enjoyment, and mental concentration. The results demonstrated that sport participation exerted a significant positive effect on attentional control, both directly and indirectly through all three flow dimensions, confirming a pattern of partial mediation. These findings highlight that the enhancement of attentional control through sport is not merely a physiological outcome of exercise but a psychological process grounded in optimal engagement, intrinsic motivation, and focused immersion.

### 5.2 Direct effect of sport participation on attentional control

The research results confirmed that badminton participation has a significant positive direct effect on attentional control, supporting Hypothesis 1 (*H1*). This finding is consistent with previous research, which shows that regular participation in sports requiring rapid reactions and sustained attention can enhance cognitive control processes and improve the effective allocation of attention resources (Noguera et al., 2019; Rahimi et al., 2022).

From the perspective of executive function theory, attentional control is a core component of executive functions, enabling individuals to inhibit distractions, maintain focus, and flexibly adjust attention when task demands change (Miyake and Friedman, 2012). Badminton participation involves continuous perceptual monitoring, decision-making, and motor coordination in a dynamic competitive environment. This situation requires participants to repeatedly engage in executive control processes, thereby improving their ability to regulate attentional focus. Frequent exposure to these cognitive demands during play helps strengthen neural circuits associated with selective attention and cognitive inhibition, thereby enhancing attentional control.

This result can also be explained through the attention resource allocation theory, which posits that individuals possess limited attention resources that must be effectively distributed across concurrent tasks (Bruya and Tang, 2018). Badminton requires athletes to quickly process spatial and temporal cues, anticipate opponents’ movements, and make real-time strategic adjustments. These repeated attentional demands promote more efficient allocation and regulation of limited attention resources. Over time, this adaptive engagement helps develop a more stable and flexible attention control system.

Consistent with previous research, this study suggests that sports participation is not only a physical activity but also a cognitive training process that enhances attentional mechanisms through repeated perceptual-cognitive interactions (Leischik et al., 2021; Mao et al., 2024). This direct effect provides empirical evidence on how sports participation, especially in cognitively demanding activities such as badminton, enhances attentional control, thereby supporting the theoretical connection between physical activity and cognitive regulation functions.

### 5.3 Mediating role of flow experience

#### 5.3.1 Perceived control as a mediator

The mediating effect of perceived control between badminton participation and attentional control was statistically significant, supporting Hypothesis 2 (*H2*). This finding is consistent with the core principles of flow theory, which emphasizes that when the challenge of an activity matches an individual’s skills, they experience a sense of control (Fong et al., 2015). In this context, individuals experience a balance between environmental demands and personal capabilities, leading to feelings of mastery and competence. In badminton participation, athletes are continually required to regulate pace, predict shuttlecock trajectories, and coordinate fine motor movements, all of which enhance their sense of control over performance outcomes. This dynamic balance between challenge and ability promotes a stable psychological state, helping to maintain focus and effectively manage attention resources.

From the perspective of self-determination theory, perceived control reflects the satisfaction of the basic psychological need for “competence”—the feeling that one can effectively interact with the environment and influence outcomes (Ryan and Deci, 2000). When individuals believe they can manage the complexity of the sport, intrinsic motivation increases, deepening psychological engagement. This intrinsic motivation further promotes attentional persistence and cognitive regulation, as individuals are more willing to invest mental effort and maintain focus on task-relevant stimuli.

The results indicate that perceived control, as a key psychological pathway, helps individuals achieve optimal attentional regulation by enhancing agency and competence during badminton participation. This finding extends previous research, suggesting that perceived control not only contributes to task performance but also reinforces executive functions by helping individuals more effectively allocate attention in demanding contexts.

#### 5.3.2 Perceived enjoyment as a mediator

The mediating role of perceived enjoyment was also confirmed, supporting Hypothesis 3 (*H3*). This finding reflects the emotional component of the flow experience, emphasizing the intrinsic pleasure and satisfaction derived from full engagement in an activity. According to flow theory, when individuals are deeply absorbed in a task, perceived enjoyment emerges, and they experience an autotelic state in which the activity itself becomes rewarding (Fong et al., 2015). In the context of badminton, the rhythmic coordination of movements, rapid exchanges, and successful rallies evoke strong positive emotions, which reinforce intrinsic motivation and sustain attention during performance.

This result can also be further explained through the broaden-and-build theory of positive emotions, which posits that positive emotional states broaden an individual’s thought-action repertoire and enhance cognitive flexibility (Fredrickson, 2004). Enjoyment stimulates exploratory and creative cognitive processes, allowing individuals to allocate attention resources more effectively and remain engaged with task-relevant cues. In this way, perceived enjoyment acts as an emotional amplifier, enhancing attentional stability and resistance to distraction. The positive emotions experienced during play help reduce cognitive fatigue and promote the continuity of the flow state, thereby improving executive regulation efficiency.

From the perspective of self-determination theory, perceived enjoyment is closely related to intrinsic motivation, which drives individuals to engage in activities for the inherent satisfaction rather than external rewards (Ryan and Deci, 2000). When badminton participants experience enjoyment, they are more likely to maintain sustained cognitive involvement, voluntarily regulate attention, and remain immersed in the activity. This intrinsically motivated engagement strengthens attentional control mechanisms by enhancing voluntary focus and reducing interference from irrelevant stimuli.

Overall, perceived enjoyment represents a way in which sports participation enhances attentional control through an emotional pathway. The integration of flow experience and positive emotion theories suggests that enjoyment not only enhances subjective well-being but also promotes the effective use of cognitive resources.

#### 5.3.3 Mental concentration as a mediator (*H4*)

The mediating effect of mental concentration between badminton participation and attentional control was significant, supporting Hypothesis 4 (*H4*). This dimension captures the cognitive core of the flow experience, representing a state of heightened focus and immersion during an activity. According to flow theory, mental concentration reflects the process of narrowing the scope of attention to task-relevant cues, during which external stimuli are effectively filtered out, and cognitive resources are fully engaged in the activity (Fong et al., 2015). In badminton, the continuous perception-action coupling—tracking the shuttlecock trajectory, predicting the opponent’s movements, and executing precise shots—requires sustained cognitive engagement. This high level of concentration enables participants to maintain acute awareness of the dynamic context, thereby promoting greater attentional stability and precision.

From the perspective of attention resource allocation theory, mental concentration serves as an adaptive mechanism that helps manage limited cognitive capacity (de Fockert, 2013). By selectively focusing resources on task-critical information and inhibiting irrelevant distractions, individuals in a concentrated state can achieve more efficient cognitive processing. This focused engagement reduces the cost of attention switching and enhances the coordination between perceptual monitoring and motor execution. Therefore, the mental concentration experienced in badminton helps train individuals to allocate and sustain attention under high-demand conditions, strengthening attentional control.

Within the framework of executive function theory, mental concentration also reflects enhanced top-down control over cognitive processes (Hopfinger and Slotnick, 2020). The regulation of attentional focus in complex sports environments involves executive functions such as inhibition, working memory updating, and cognitive flexibility (Brimmell et al., 2022). Repeated engagement in these processes during gameplay strengthens executive pathways, improving an individual’s overall attentional regulation capacity.

In summary, mental concentration, as a key cognitive mediator, links the behavioral demands of badminton participation with the development of attentional control. By promoting deep attentional immersion and minimizing interference from irrelevant stimuli, this dimension of flow allows participants to maintain a stable, goal-directed cognitive state. These findings further demonstrate that the cultivation of attentional control is not only achieved through the frequency of sports participation but is also enhanced by the depth and quality of attentional involvement experienced during the activity.

### 5.4 Integrated discussion of the mediation model

The integrated analysis of the mediation model shows that the three dimensions of flow experience—perceived control, perceived enjoyment, and mental concentration—collectively mediate the relationship between badminton participation and attentional control. These results suggest that the process of enhancing attentional control through sports participation is not linear, but instead is achieved through a multidimensional psychological mechanism encompassing both cognitive regulation and emotional experience.

From the perspective of flow theory, this pattern indicates that optimal psychological functioning is achieved when individuals simultaneously experience a sense of control, intrinsic enjoyment, and deep concentration during physical activities (Fong et al., 2015). The interaction among these dimensions creates a self-reinforcing state, where perceived control stabilizes focus, enjoyment sustains engagement, and concentration intensifies cognitive immersion. Together, they form an integrated flow system that supports efficient attentional allocation and the inhibition of irrelevant stimuli. This synergy exemplifies the holistic nature of flow, in which emotional, motivational, and cognitive processes coalesce to produce heightened attention and performance.

From the perspective of attention resource allocation theory, this integration reflects the optimized use of limited cognitive resources (de Fockert, 2013). Perceived control ensures efficient attentional focus by reducing uncertainty; perceived enjoyment maintains resource investment through intrinsic motivation; and mental concentration maximizes the precise deployment of resources by filtering distractions. The coordinated functioning of these mechanisms leads to more stable and flexible attentional regulation in high-demand sports contexts.

Furthermore, in line with executive function theory, the integrated mediation model indicates that flow experience serves as a natural cognitive training mechanism (Hopfinger and Slotnick, 2020). By continuously engaging in inhibitory control, cognitive flexibility, and goal maintenance within the sports environment, flow-based participation helps reinforce the executive pathways that support attentional control. Therefore, the flow state is not just an accompaniment to effective performance but actively cultivates the executive structures responsible for attentional regulation.

### 5.5 Limitations and future research directions

Although the present study provides empirical support for the mediating role of flow experience in the relationship between badminton participation and attentional control, several limitations should be acknowledged, and directions for future research are proposed accordingly.

First, the study employed a cross-sectional design, which limits the ability to infer causality between variables. Although the structural model was theoretically grounded and statistically robust, causal relationships among sport participation, flow experience, and attentional control cannot be conclusively established. Future research should employ longitudinal or experimental designs to verify the temporal sequence and causal direction of these effects. Intervention-based studies, in which individuals engage in structured badminton programs, would provide stronger evidence of the causal mechanisms linking physical activity and attentional control.

Second, the sample consisted exclusively of college students, which may restrict the generalizability of the findings. University students typically share similar cognitive and motivational characteristics, and their flow experiences may differ from those of other age or professional groups. Future studies should include broader and more diverse samples, such as adolescents, athletes at different competitive levels, or adult populations engaged in recreational sports, to test the stability and universality of the observed relationships. Additionally, future research could explore the potential differences in flow experience and attentional control across different levels of athletic training and physical activity frequency. Stratifying samples based on these factors would enhance the depth and complexity of the findings and may reveal how different levels of sport engagement influence attention control.

In addition, although reports basic demographic information for the present sample (gender, academic year, and major), more fine-grained indicators of academic workload (e.g., weekly study hours, perceived academic pressure) and detailed badminton training history (e.g., years of systematic training, competitive level, formal coaching experience) were not collected. As a result, the potential confounding or moderating effects of these factors on the relationship between badminton participation, flow experience, and attentional control could not be examined in the current study. Future research should incorporate more detailed measures of academic workload and sport-specific training background to better characterize the sample and to test whether these factors shape or condition the observed associations.

Third, all measures relied on self-reported questionnaires, which may introduce common method bias and subjective bias despite the statistical checks conducted. Future research should incorporate multi-method assessments, including behavioral indicators of attention (e.g., Stroop or Flanker tasks), physiological measures (e.g., EEG or eye-tracking), or real-time experience sampling to capture the dynamic characteristics of flow and attention in sport contexts. These objective measures would increase the reliability and validity of the findings and provide a more comprehensive understanding of how flow experience influences attention control.

Fourth, the present research focused exclusively on badminton, a sport characterized by high reactivity and cognitive demand. While this focus offers a clear theoretical framework, it also limits the ecological validity of the findings. Future work should compare different types of physical activities—such as endurance, esthetic, and team-based sports—to determine whether the mediating mechanisms of flow experience operate similarly across varying motor and cognitive demands. This comparative approach would provide a more nuanced understanding of how different sport types influence attentional control.

Finally, future studies could further explore contextual and personality moderators that may influence the strength of the observed relationships, such as individual differences in trait self-control, motivation orientation, or prior sport experience. Examining these factors would deepen understanding of how individual and environmental characteristics shape the formation and effects of flow experience. Moreover, investigating how cognitive load from daily activities or academic pressures might interact with sport participation could offer insights into how external stressors may affect attentional control and flow experiences.

In summary, while the present study advances understanding of the psychological mechanisms linking sport participation and attentional control, future research employing longitudinal, multi-method, and comparative designs will be essential to refine and extend these conclusions across broader contexts and populations.

## 6 Theoretical and practical implications

### 6.1 Theoretical implications

The present study contributes to the theoretical understanding of how sport participation facilitates attentional control by identifying flow experience as a multidimensional mediating mechanism. These findings extend existing frameworks in sport psychology and cognitive science through three major theoretical implications.

First, the study extends the application of flow theory to high-reactivity and cognitively demanding sports such as badminton. Previous research on flow has primarily focused on endurance or esthetic sports, emphasizing emotional enjoyment and performance optimization. By contrast, this study demonstrates that flow in badminton—a sport requiring continuous perceptual monitoring and rapid decision-making—also functions as a critical mechanism for enhancing cognitive regulation. The confirmation of the mediating effects of perceived control, perceived enjoyment, and mental concentration reveals that the flow state is not merely an affective experience but also a structured cognitive–motivational system that influences attentional processes. This extends Csikszentmihalyi’s conceptualization of flow by emphasizing its role in facilitating executive regulation and attentional stability in dynamic task environments.

Second, the findings provide empirical validation of attention resource allocation theory within the context of sport participation. The results indicate that individuals who experience flow allocate limited cognitive resources more efficiently by filtering distractions and sustaining focus on task-relevant cues. The three dimensions of flow correspond to different mechanisms within this framework: perceived control optimizes resource allocation through expectancy and mastery beliefs; perceived enjoyment maintains motivational engagement that prevents attentional depletion; and mental concentration enhances the precision and stability of resource deployment. Together, these mechanisms illustrate how optimal attentional control arises from the dynamic coordination of cognitive, emotional, and motivational processes during physical activity.

Third, this research integrates the principles of executive function theory with the flow experience framework, providing a unified explanation for how sport participation strengthens higher-order cognitive control. The repeated engagement in decision-making, inhibitory control, and situational adaptation inherent in badminton reinforces the neural pathways responsible for attentional regulation. Flow experience, as shown in this study, serves as the psychological context that enables such engagement to occur efficiently and sustainably. This integration underscores that attentional control improvement through sport is not solely the result of repetitive physical execution but emerges from the quality of cognitive involvement and motivational absorption that characterizes the flow state.

### 6.2 Practical implications

The findings of this study also offer several important practical implications for physical education, sport psychology interventions, and cognitive enhancement among college students. By clarifying how flow experience mediates the relationship between sport participation and attentional control, the results provide empirical guidance for designing sport-based programs that optimize both psychological engagement and cognitive outcomes.

First, the study underscores the importance of designing sport activities that promote flow experience. In the context of badminton and similar reactive sports, instructors and coaches should seek to maintain an optimal balance between task challenge and player skill. Providing appropriately graded difficulty levels and immediate feedback can strengthen perceived control, enabling participants to experience a sense of mastery and competence. This balanced challenge–skill structure not only sustains motivation but also cultivates attentional engagement throughout the activity.

Second, the findings highlight the role of positive emotion and intrinsic motivation in enhancing attention regulation. Creating a supportive and autonomy-oriented environment—one that emphasizes enjoyment, voluntary participation, and self-directed improvement—can foster perceived enjoyment, which in turn maintains attentional persistence and reduces cognitive fatigue. Sport educators should focus on fostering intrinsic goals rather than emphasizing competitive outcomes alone, thereby transforming participation into a psychologically rewarding experience that strengthens cognitive focus.

Third, the dimension of mental concentration provides practical insight for sport psychology and cognitive training. Structured interventions that train individuals to sustain focus under pressure, such as attentional cueing, mindfulness-based attention exercises, or concentration drills integrated into badminton practice, can enhance the depth of mental engagement. These interventions help participants internalize attentional control strategies that can transfer to learning and other cognitive tasks beyond the sporting context.

Furthermore, the study’s findings can inform university-based mental health and educational initiatives. By integrating structured physical activity programs that facilitate flow states, institutions can address the prevalent issues of attention lapses, academic fatigue, and psychological stress among students. Encouraging participation in cognitively engaging sports like badminton can serve as a preventive and developmental strategy to promote both cognitive functioning and emotional well-being.

## 7 Conclusion

This study explored the psychological mechanisms linking badminton participation with attentional control among college students, focusing on the mediating role of flow experience and its three dimensions—perceived control, perceived enjoyment, and mental concentration. The results highlighted that sport participation significantly influenced attentional control both directly and indirectly through all three flow dimensions, demonstrating partial mediation. These findings suggest that enhancing attentional control through sport is not solely a physiological outcome but a psychological process involving optimal engagement, intrinsic motivation, and deep concentration.

Theoretically, the study expands flow theory to high-reactivity sports, supports attention resource allocation theory within a sport context, and integrates executive function perspectives to understand cognitive regulation through physical activity. Practically, the model offers valuable insights for developing sport-based interventions aimed at cultivating flow states, which can be applied to enhance attention, learning efficiency, and psychological well-being in both educational and sport psychology settings.

## Data Availability

The original contributions presented in the study are included in the article/supplementary material, further inquiries can be directed to the corresponding author.
